# A Mathematical Model of Vaccinations Using New Fractional Order Derivative

**DOI:** 10.3390/vaccines10121980

**Published:** 2022-11-22

**Authors:** Asma, Mehreen Yousaf, Muhammad Afzaal, Mahmoud H. DarAssi, Muhammad Altaf Khan, Mohammad Y. Alshahrani, Muath Suliman

**Affiliations:** 1 Department of Mathematics, COMSATS University Islamabad, Sahiwal Campus, Sahiwal 57000, Pakistan; 2 DHQ Teaching Hospital, Sahiwal 57000, Punjab, Pakistan; 3 Lahore General Hospital, Lahore 54000, Punjab, Pakistan; 4 Department of Basic Sciences, Princess Sumaya University for Technology, Amman 11941, Jordan; 5 Institute for Ground Water Studies, Faculty of Natural and Agricultural Sciences, University of the Free State, Bloemfontein 9301, South Africa; 6 Department of Mathematics, Faculty of Science and Technology Universitas Airlangga, Surabaya 60115, Indonesia; 7 Department of Clinical Laboratory Sciences, College of Applied Medical Sciences, King Khalid University, P.O. Box 61413, Abha 9088, Saudi Arabia

**Keywords:** generalized fractional derivative, real cases, backward bifurcation, numerical results

## Abstract

Purpose: This paper studies a simple SVIR (susceptible, vaccinated, infected, recovered) type of model to investigate the coronavirus’s dynamics in Saudi Arabia with the recent cases of the coronavirus. Our purpose is to investigate coronavirus cases in Saudi Arabia and to predict the early eliminations as well as future case predictions. The impact of vaccinations on COVID-19 is also analyzed. Methods: We consider the recently introduced fractional derivative known as the generalized Hattaf fractional derivative to extend our COVID-19 model. To obtain the fitted and estimated values of the parameters, we consider the nonlinear least square fitting method. We present the numerical scheme using the newly introduced fractional operator for the graphical solution of the generalized fractional differential equation in the sense of the Hattaf fractional derivative. Mathematical as well as numerical aspects of the model are investigated. Results: The local stability of the model at disease-free equilibrium is shown. Further, we consider real cases from Saudi Arabia since 1 May–4 August 2022, to parameterize the model and obtain the basic reproduction number $R_{0}^{v}\approx 2.92$. Further, we find the equilibrium point of the endemic state and observe the possibility of the backward bifurcation for the model and present their results. We present the global stability of the model at the endemic case, which we found to be globally asymptotically stable when $R_{0}^{v}>1$. Conclusion: The simulation results using the recently introduced scheme are obtained and discussed in detail. We present graphical results with different fractional orders and found that when the order is decreased, the number of cases decreases. The sensitive parameters indicate that future infected cases decrease faster if face masks, social distancing, vaccination, etc., are effective.

## 1. Introduction

Mathematical models are recognized as crucial in epidemiology for understanding the dynamics of diseases and making predictions about their long-term behavior. With the passage of time and the emergence of new infectious diseases in humans populations, mathematical models have been used to determine the peak infection curve, the days to eradication, and the number of possible future cases. In the case of the coronavirus disease, the early prediction of the peak of the infection, the basic reproduction number, and the possible elimination of the disease, have been shown by simple types of SIR, etc. The emergence of the coronavirus infection resulted in a huge number of illnesses and fatalities globally at a time when many countries were experiencing a financial crisis. According to reports, many infected cases in Saudi Arabia resulted in death. To date, the kingdom has recorded 9271 deaths and 812,093 total cases [1]. Following the implementation of the World Health Organization’s (WHO’s) recommendations by the Saudi government, the number of new cases was found to be lower in comparison to recent instances. It is commonly known that the coronavirus outbreak is continuing in many nations throughout the world, with two, three, or more waves. In Saudi Arabia, three waves of COVID-19 have been previously detected, and the fourth wave is currently ongoing, with the expectation that it will terminate in the near future. In comparison to the past waves of infections, the present cohort of patients will not yield as many infected cases.

Mathematical models are used to study biological and physical problems widely in the literature to understand the complicated nonlinear process of nonlinear problems see [2,3,4,5]. Both integer and non-integer order problems have been studied in the literature in the recent past, and some recommendations about the disease control controls have been given; see [6,7,8,9,10]. For applications of mathematical models to study COVID-19’s dynamics and their possible controls, one can see [11,12,13,14,15,16,17,18,19,20]. For instance, the COVID-19 infection model incorporating the lockdown phenomenon has been studied in [11]. The concept of the Continuous Markov-Chain in the modeling of COVID-19 has been presented in [12]. The study of Omicron and its dynamical analysis for the second wave has been proposed in [13]. A fractional modeling approach to study SARS-CoV-2 using real cases is discussed in [14]. Modeling and control measures for COVID-19 have been discussed in [15]. The authors of [16] collected coronavirus infection cases from Ethiopia and built a mathematical model and studied their analysis. The outbreak of coronavirus infections throughout the world resulted in many people experiencing stress and tension; this impact has been used in the modeling of coronavirus by the authors of [17]. The reported cases of the coronavirus in Saudi Arabia have been considered in a mathematical study by the authors of [18]. The self-isolation study through a mathematical model of the coronavirus disease has been discussed in [19]. In another study, the authors utilized the concept of mathematical modeling to design a new mathematical model for the reported cases in Saudi Arabia [20]. Some other related work studying the COVID-19 infection can be found in the literature. In one study, the authors considered a mathematical model for the reported cases in India and established the optimal control model for its possible disease eliminations [9]. One of the good controls for COVID-19 infection by using face masks has been explored in [21]. COVID-19 infection and its coinfection with cholera has been documented in [22]. The specific applications to Turkish data through a mathematical model are considered in [23]. A COVID-19 infection model with waning immunity has been discussed in [24]. In [25], the authors considered the mathematical analysis of COVID-19 infection. The coinfection model of dengue and COVID-19 from a clinical perspective and the future challenges have been addressed in [26]. The diffusion process and its relation to the modeling of COVID-19 have been investigated in [27]. A delay differential equations model to study COVID-19 is suggested in [28]. For some other research papers regarding COVID-19, we refer the readers to see [29,30,31,32]. The authors of [29] utilize the real data of COVID-19 infection in Sri Lanka and present results regarding infection minimization. The authors of [30] present a mathematical model to predict future cases of COVID-19 infection. A mathematical model has been constructed to study the second wave of COVID-19 in Italy in [31]. A mathematical model has been designed and analyzed using the cases in Thailand [32].

Vaccines can be regarded as a useful control for any viral disease. For example, the authors proposed a vaccination model with treatment to study the epidemic disease in [33]. In the past, infections of many diseases have been controlled or reduced using vaccination, such as Polio, Hepatitis B, Flu (Influenza), Rubella, Hepatitis A, Tetanus, and many more. With the passage of time and the emergence of new infectious diseases that humans society faces, researchers are always looking for safe and effective vaccines. In this regard, various vaccines around the world have been introduced by different researchers and found effective against the coronavirus. COVID-19 vaccines provide immunity to individuals and protect them from future illness. It is safe and most people can use it without any fear [34,35,36,37].

This paper investigates the dynamics of the coronavirus infection in Saudi Arabia using recently reported cases through the fractional-order vaccination model. We use the recent cases of the fourth wave in Saudi Arabia and implement a mathematical model first in the integer order and then extend it to the generalized order model. The model is directly fitted to the cases in which the vaccine is present and studies the equilibrium points analysis. We observe the possibility of a backward bifurcation phenomenon, where the disease-free equilibrium coexists with the endemic state and hence the global asymptotical stability of the disease-free equilibrium does not exist. We divide the work section-wise: Section 2 gives details of the newly fractional derivative considered by Hattaf [38]. Further, it discusses the formulation of the problem and further extends the model into the fractional order system. Furthermore, we study the equilibrium points, the basic reproduction, backward bifurcation, and the local asymptotical stability of the disease-free case and explain the algorithm for the numerical simulation of the fractional model. In Section 3, we discuss the graphical results and present results regarding disease controls. The results are summarized briefly in Section 4.

## 2. Materials and Methods

### 2.1. Background Results

Here, we give some important results regarding fractional calculus and its onward use in the results of the paper.

**Definition** **1.** *Let $q\in \left[0,1\right),q_{1},q_{2}>0$, and $g\in H^{1}\left(l_{1},l_{2}\right)$. Then, for the function g(t) with another function ϕ(t) for the order q, the generalized fractional derivative in the sense of Caputo is given by [38]:*

(1) $$\begin{array}{c} {}^{C}D_{l_{1},t,\phi }^{q,q_{1},q_{2}}g\left(t\right)=\frac{N(q)}{1-q}\frac{1}{\phi (t)}\int_{l_{1}}^{t}E_{q_{1}}\left[-\mu_{q}{\left(t-\xi \right)}^{q_{2}}\right]\frac{d}{d\xi }\left(\phi g\right)\left(\xi \right)d\xi , \end{array}$$

*where $\phi \in C^{1}\left(l_{1},l_{2}\right),\phi ,\phi^{\prime }>0$ on [a,b],N(q), defining the normalization function and satisfying $N\left(0\right)=N\left(1\right)=1,\mu_{q}=q/1-q$, and $E_{q_{1}}\left(t\right)=\sum_{k=0}^{+\infty }\frac{t^{k}}{\Gamma (q_{1}k+1)}$, denotes the Mittag–Leffler function of the parameter $q_{1}$.*

Below, for Definition (1), we can write the corresponding fractional integral as

**Definition** **2** ([38])**.** *For the newly fractional derivative $D_{l_{1},\phi }^{q,q_{1}}$, the corresponding fractional integral can be expressed as*

(2) $$\begin{array}{c} D_{l_{1},\phi }^{q,q_{1}}g\left(t\right)=\frac{1-q}{N(q)}g\left(t\right)+\frac{q}{N(q)}RL_{J_{l_{1},\phi }^{q_{1}}}g\left(t\right) \end{array}$$

*where ${}^{RL}I_{l_{1},\phi }^{q_{1}}$ of order $q_{1}$ denotes the standard weighted Riemann–Liouville fractional integral and is defined by*

(3) $$\begin{array}{c} {}^{RL}J_{l_{1},\phi }^{q_{1}}g\left(t\right)=\frac{1}{\Gamma (q_{1})}\frac{1}{\phi (t)}\int_{l_{1}}^{t}{\left(t-\xi \right)}^{q_{1}-1}\phi \left(\xi \right)g\left(\xi \right)d\xi . \end{array}$$

**Theorem** **1.** ([39])**.** *Suppose y=0 is an equilibrium point of*

(4) $$D_{0,\phi }^{q,q_{1}}y\left(t\right)=f\left(y\left(t\right)\right)$$

*and V(y) is a continuously differentiable function in a neighborhood $U\in R^{n}$ of the origin holds the conditions below:*

*(i)*  *V(0)=0 and V(y)>0 for all y∈U∖{0};*
*(ii)*  *$D_{0,\phi }^{q,q_{1}}V\left(y\right)\leq 0$ for all y∈U∖{0}.*

*Then, y=0 is stable.*

### 2.2. Model Formulation

We consider an SVIR model and denote its total population by N(t). The model consists of four components: the healthy individuals that have the ability to become infected after close contact with infected COVID-19 people is shown by S(t); individuals that are vaccinated are given by V(t); individuals that are infected are given by I(t); and those recovered from infection of COVID-19 or vaccination are given by R(t). We write N(t)=S(t)+V(t)+I(t)+R(t). The population of healthy individuals is obtained through the birth rate Λ, while the natural mortality rate in each compartment is given by μ. Healthy individuals become infected when they have close contact with infected people, and hence the route of the transmission is βSI/N, while vaccinated individuals after close contact with infected people are shown through the route $\beta_{1}VI/N$. The portion of healthy individuals to be vaccinated is shown by ω. The vaccinated and the infected individuals are recovered, respectively, by the rate $\gamma_{1}$ and γ. The disease mortality of the COVID-19 infected people in the infected compartment is given by $d_{1}$. With these assumptions, the COVID-19 model with vaccination is given by the following nonlinear differential equations:

(5) $$\begin{array}{c} \left\{\begin{array}{c} \frac{dS}{dt}=\Lambda -\frac{\beta SI}{N}-\left(\mu +\omega \right)S,\;\\ \frac{dV}{dt}=\omega S-\frac{\beta_{1}VI}{N}-\left(\mu +\gamma_{1}\right)V,\;\\ \frac{dI}{dt}=\frac{\beta SI}{N}+\frac{\beta_{1}VI}{N}-\left(\gamma +\mu +d_{1}\right)I,\;\\ \frac{dR}{dt}=\gamma_{1}V+\gamma I-\mu R, \end{array}\right. \end{array}$$

with the non-negative initial conditions

$$\begin{array}{c} S\left(0\right)\geq S_{0},V\left(0\right)\geq V_{0},I\left(0\right)\geq I_{0},R\left(0\right)\geq R_{0}. \end{array}$$

We consider the following biologically feasible region for the model (5),

$$\begin{array}{c} \Gamma =\left\{\left(S,V,I,R\right)\in R_{+}^{4}:S,V,I,R\geq 0,\;and\;N\leq \Lambda /\mu \right\}, \end{array}$$

which is positively invariant for any trajectory of the system for an initial condition, which will remain in Γ for every time t≥0. Therefore, the region is positively invariant, and its dynamical results can be studied within Γ. It can be observed from model (5) that the equation *R* can be eliminated without any loss of generality, as it does not appear in the rest of the equation. The results of *R* can be easily obtained using the relation R=N−S−V−I. Using this fact, in the following, we focus our study to analyze the fractional model without the last equation. There are no transmission rates from equations *R* to the rest of the equations, so one can ignore and reduce it, while the results of recovery cases can be obtained using the equation R=N−S−V−I.

### 2.3. A Fractional Model

We apply the recently introduced fractional derivative by Hattaf given in Definition 1 to our model (5) and obtain the following generalized fractional order model:

(6) $$\begin{array}{c} \left\{\begin{array}{c} {}^{H}D_{0,\phi }^{q,q_{1}}S\left(t\right)=\Lambda -\frac{\beta SI}{N}-\left(\mu +\omega \right)S,\;\\ {}^{H}D_{0,\phi }^{q,q_{1}}V\left(t\right)=\omega S-\frac{\beta_{1}VI}{N}-\left(\mu +\gamma_{1}\right)V,\;\\ {}^{H}D_{0,\phi }^{q,q_{1}}I\left(t\right)=\frac{\beta SI}{N}+\frac{\beta_{1}VI}{N}-\left(\gamma +\mu +d_{1}\right)I, \end{array}\right. \end{array}$$

and the related initial conditions

(7) $$\begin{array}{c} S\left(0\right)\geq S_{0},V\left(0\right)\geq V_{0},I\left(0\right)\geq I_{0}. \end{array}$$

### 2.4. Analysis of the Model

This section considers the mathematical results involved in the fractional order system (6). In a dynamical system, first, we obtain the possible equilibrium points of the disease model (6). In general, the models often formulated for the disease-related human population consist of two equilibrium points, the infection-free and the infected. The infection-free equilibrium can be denoted by $P_{0}$ of the model (6), which one can obtain as follows:

$$\begin{array}{c} P_{0}=\left(S^{0},V^{0},0\right)=\left(\frac{\Lambda }{\mu +\omega },\frac{\Lambda \omega }{\left(\gamma_{1}+\mu \right)\left(\mu +\omega \right)},0\right). \end{array}$$

Another important concept in disease epidemiology is the computation of the basic reproduction number. The basic reproduction tells us about the disease’s progress, and whether it can be controlled or spread within the population. For our SVIR-type model (6), we can determine this number using the last equation of the system (6) within the disease-free case $P_{0}$ and obtain the following result:

$$\begin{array}{ccc} R_{0}^{v} & = & \underset{R_{1}}{\underbrace{\frac{\beta_{1}\omega }{\left(\gamma_{1}+\mu +\omega \right)\left(\gamma +d_{1}+\mu \right)}}}+\underset{R_{2}}{\underbrace{\frac{\beta \left(\gamma_{1}+\mu \right)}{\left(\gamma_{1}+\mu +\omega \right)\left(\gamma +d_{1}+\mu \right)}}}. \end{array}$$

The basic reproduction, or in this case the vaccine reproduction number $R_{0}^{v}$, consists of two parts: the first part $R_{1}$ is associated with vaccine cases, while the other one $R_{2}$ is related to cases without vaccination. It is obvious that the vaccine reduces the basic reproduction number, as vaccines for any disease in the literature prove that vaccines are the best control of disease. We obtain the basic reproduction number with no vaccination by putting ω=0, and obtain the following:

$$\begin{array}{ccc} R_{0} & = & \frac{\beta }{\left(\gamma +d_{1}+\mu \right)}. \end{array}$$

### 2.5. Endemic Equilibria

Here, we shall investigate the endemic equilibrium of the vaccine model (6) given by $P_{1}$

(8) $$\begin{array}{c} P_{1}=\left(S,V,I\right)=\left(S^{*},V^{*},I^{*}\right) \end{array}$$

and can determined by equating ${}^{H}D_{0,\phi }^{q,q_{1}}S\left(t\right)={}^{H}D_{0,\phi }^{q,q_{1}}V\left(t\right)={}^{H}D_{0,\phi }^{q,q_{1}}I\left(t\right)=0$, and obtain the following,

(9) $$\begin{array}{c} \left\{\begin{array}{c} S^{*}=\frac{\Lambda }{\beta \lambda^{*}+\mu +\omega },\;\\ V^{*}=\frac{\omega S^{*}}{\beta_{1}\lambda^{*}+\gamma_{1}+\mu },\;\\ I^{*}=\frac{\beta \lambda^{*}S^{*}+\beta_{1}\lambda^{*}V^{*}}{\gamma +d_{1}+\mu }. \end{array}\right. \end{array}$$

Inserting (9) into the following expression, and obtain

(10) $$\begin{array}{c} \lambda^{*}=\frac{I^{*}}{N^{*}}, \end{array}$$

We obtain the following,

(11) $$\begin{array}{c} a_{0}\lambda^{2}+a_{1}\lambda +a_{2}=0, \end{array}$$

where

(12) $$\begin{array}{c} \begin{array}{c} a_{0}=\beta \beta_{1},\;\\ a_{1}=\beta \left(\gamma_{1}+\mu \right)+\beta_{1}\left(-\beta +\gamma +d_{1}+\mu +\omega \right),\;\\ a_{2}=\left(\gamma_{1}+\mu +\omega \right)\left(\gamma +d_{1}+\mu \right)\left(1-R_{0}^{v}\right). \end{array} \end{array}$$

Here, it can be seen that $a_{0}>0$ and $a_{2}$ can be positive if $R_{0}^{v}<1$, while it is negative if $R_{0}^{v}>1$. The result for the endemic equilibria can be summarized in the following form:

**Theorem** **2.** *The coronavirus model (6) has the following:*

*1.*  *There is exists a unique endemic equilibrium if $a_{2}<0⟺R_{0}^{v}>1$,*
*2.*  *There exists a unique endemic equilibrium if $a_{1}<0$ and $a_{2}=0\to R_{0}^{v}=1$,*
*3.*  *We can have two endemic equilibria if $a_{2}>0\to R_{0}^{v}<1$, $a_{1}<0$ and its related discriminant is positive*
*4.*  *Above the other cases, there is no possible equilibria.*

It is clear from the first part (i) of the Theorem 2 that we have a unique positive endemic equilibrium whenever $R_{0}^{v}>1$. Further, the third part of Theorem 2 tells us about the occurrence of the phenomenon of backward bifurcation in the COVID-19 infection model (6). This means that disease-free equilibrium coexists with endemic equilibrium, and the model will not be globally asymptotically stable. In such a case, the disease will persist in the population for a long time and need vaccination and other control measures for its elimination and control. To achieve the mathematical expression and its graphical result, we set the discriminant $a_{1}^{2}-4a_{0}a_{2}=0$ and then solve further for the critical values of $R_{0}^{v}$ denoted by $R_{c}$, which is given by

(13) $$\begin{array}{c} R_{c}=\sqrt{1-\frac{a_{1}^{2}}{4a_{0}\left(\gamma_{1}+\mu +\omega \right)\left(\gamma +d_{1}+\mu \right)}}. \end{array}$$

**Figure 1:**
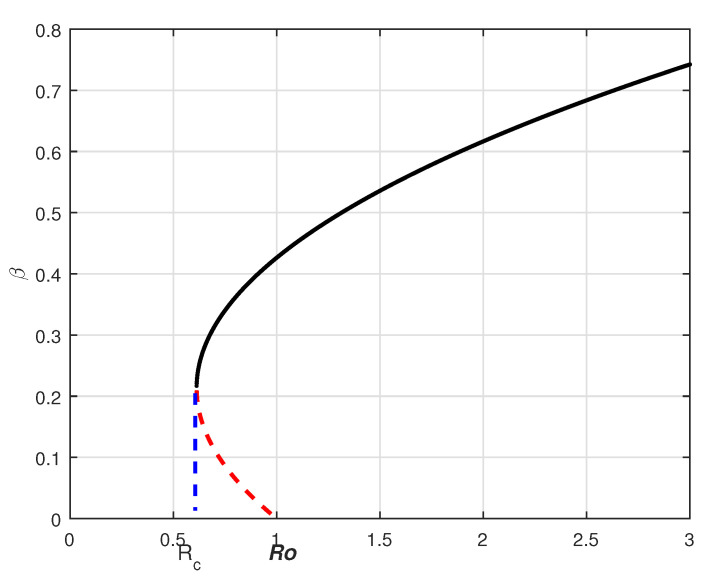
Backward bifurcation graph for the system (6).

Therefore, the backward bifurcation may occur for the values of $R_{0}^{v}$ such that $R_{c}<R_{0}^{v}<1$. Consider the listed value Λ=1273.94, β=0.6, $\beta_{1}=0.2$, γ=0.05, $\gamma_{1}=0.04$, μ=1/(74.87×365), $d_{1}=0.024$ and ω=0.15. The related bifurcation plot is given in Figure 1. In Figure 1, one can see that β is the bifurcation parameter that can cause the backward bifurcation. In such cases, the model may or may not be globally asymptotically stable at the disease-free equilibrium.

### 2.6. Stability Analysis

The stability analysis of Model (5) can be studied for the disease-free case. We present these results in the following theorem:

**Theorem** **3.** *The fractional-order SVIR model is locally asymptotically stable, provided that $R_{0}^{v}<1$.*

**Proof.**  We have the Jacobian matrix of the system (6) evaluated for the disease-free case $P_{0}$, and it is given by

$$\begin{array}{c} J\left(P_{0}\right)=\left(\begin{array}{ccc} -(\mu +\omega ) & 0 & -\frac{\beta S^{0}}{S^{0}+V^{0}}\\ \omega & -(\mu +\gamma_{1}) & -\frac{\beta_{1}V^{0}}{S^{0}+V^{0}}\\ 0 & 0 & \frac{\beta S^{0}}{S^{0}+V^{0}}-\left(\gamma +\mu +d_{1}\right)+\frac{\beta_{1}V^{0}}{S^{0}+V^{0}} \end{array}\right). \end{array}$$

The Jacobian matrix $J(P_{0})$ can be expanded, and we can obtain the eigenvalues as follows: $\lambda_{1}=-\left(\mu +\omega \right)<0$, $\lambda_{2}=-\left(\gamma_{1}+\mu \right)<0$ and $\lambda_{3}=-\left(\gamma +d_{1}+\mu \right)\left(1-R_{0}^{v}\right)$. The first two eigenvalues are obviously negative, while the third eigenvalue can be negative if $R_{0}^{v}<1$. Therefore, all the roots of the Jacobian matrix contain negative real parts, so the fractional-order system (6) at the disease-free equilibrium point $P_{0}$ is locally asymptotically stable provided that $R_{0}^{v}<1$. □

#### Global Stability

To show the global stability of the model at $P_{0}$ when $R_{0}^{v}\leq 1$, we construct the Lyapunove function given by

$$\begin{array}{c} V(I)=I. \end{array}$$

We have

$$\begin{array}{c} \begin{array}{cc} {}^{H}D_{0,\phi }^{q,q_{1}}V\left(I\right) & ={}^{H}D_{0,\phi }^{q,q_{1}}I,\;\\ & \leq \frac{\beta SI}{N}+\frac{\beta_{1}VI}{N}-\left(\gamma +\mu +d_{1}\right)I,\;\\ & \leq \left(\gamma_{1}+\mu +d_{1}\right)\left(1-R_{0}^{v}\right)I. \end{array} \end{array}$$

Therefore, the above result can be stated as follows:

**Theorem** **4.** *The SVIR model is globally asymptotically stable if $R_{0}^{v}\leq 1$.*

### 2.7. Global Stability at Endemic State

We need the following results in the proof of the following theorem, with the assumption βSI≤N and $\beta_{1}VI\leq N$:

$$\begin{array}{c} \begin{array}{ccc} \Lambda & = & \beta S^{*}I^{*}+\mu S^{*}+\omega S^{*},\;\\ \frac{\omega S^{*}-\beta_{1}V^{*}I^{*}}{I^{*}} & = & (\mu +\gamma ),\;\\ \left(\gamma +\mu +d_{1}\right) & = & \frac{\beta S^{*}I^{*}+\beta_{1}V^{*}I^{*}}{I^{*}}, \end{array} \end{array}$$

**Theorem** **5.** *The SVIR epidemic model is globally asymptotically stable if $R_{0}^{v}>1$.*

**Proof.**  We define the Lyapunove function given by

(14) $$\begin{array}{c} L\left(S,V,I\right)=S^{*}\Psi \left(\frac{S}{S^{*}}\right)+V^{*}\Psi \left(\frac{V}{V^{*}}\right)+I^{*}\Psi \left(\frac{I}{I^{*}}\right), \end{array}$$

where Ψ(y)=y−1−lny, for y>0. It is clear that Ψ(y) attains its global minimum at y=1 and Ψ(1)=0. Therefore, Ψ(y)≥0 for every y>0. Thus, L(S,V,I)≥0 with $L(S^{*},V^{*},I^{*})=0$. Applying the Corollary 2 given in [40], we have

(15) $$\begin{array}{c} {}^{H}D_{0,\phi }^{q,q_{1}}L\left(t\right)\leq \left(1-\frac{S^{*}}{S}\right){}^{H}D_{0,\phi }^{q,q_{1}}S+\left(1-\frac{V^{*}}{V}\right){}^{H}D_{0,\phi }^{q,q_{1}}V+\left(1-\frac{I^{*}}{I}\right){}^{H}D_{0,\phi }^{q,q_{1}}I. \end{array}$$

Using the equation from system (6) into (15), and calculating the terms of Equation (15), we have

(16) $$\begin{array}{c} \begin{array}{ccc} \left(1-\frac{S^{*}}{S}\right){}^{H}D_{0,\phi }^{q,q_{1}}S & = & \left(1-\frac{S^{*}}{S}\right)\left[\Lambda -\beta SI-\left(\mu +\omega \right)S\right],\;\\ & = & \left(1-\frac{S^{*}}{S}\right)\left[\beta S^{*}I^{*}+\mu S^{*}+\omega S^{*}-\beta SI-\mu S-\omega S\right],\;\\ & = & \beta S^{*}I^{*}\left(1-\frac{S^{*}}{S}\right)\left(1-\frac{SI}{S^{*}I^{*}}\right)+\mu S^{*}\left(1-\frac{S^{*}}{S}\right)\left(1-\frac{S}{S^{*}}\right)\;\\ & & +\omega S^{*}\left(1-\frac{S^{*}}{S}\right)\left(1-\frac{S}{S^{*}}\right),\;\\ & = & \beta S^{*}I^{*}\left(1-\frac{S^{*}}{S}-\frac{SI}{S^{*}I^{*}}+\frac{I}{I^{*}}\right)+\mu S^{*}\left(2-\frac{S^{*}}{S}-\frac{S}{S^{*}}\right)\;\\ & & +\omega S^{*}\left(2-\frac{S^{*}}{S}-\frac{S}{S^{*}}\right). \end{array} \end{array}$$

(17) $$\begin{array}{c} \begin{array}{ccc} \left(1-\frac{V^{*}}{V}\right){}^{H}D_{0,\phi }^{q,q_{1}}V & = & \left(1-\frac{V^{*}}{V}\right)\left[\omega S-\beta_{1}VI-\left(\mu +\gamma_{1}\right)V\right],\;\\ & = & \left(1-\frac{V^{*}}{V}\right)\left[\omega S-\beta_{1}VI-\left(\frac{\omega S^{*}}{V^{*}}-\frac{\beta_{1}I^{*}V^{*}}{V^{*}}\right)V\right],\;\\ & = & \omega S^{*}\left(1-\frac{V}{V^{*}}+\frac{S}{S^{*}}-\frac{SV^{*}}{VS^{*}}\right)\;\\ & & +\beta_{1}V^{*}I^{*}\left(\frac{V}{V^{*}}-1-\frac{VI}{V^{*}I^{*}}+\frac{I}{I^{*}}\right) \end{array} \end{array}$$

(18) $$\begin{array}{c} \begin{array}{ccc} \left(1-\frac{I^{*}}{I}\right){}^{H}D_{0,\phi }^{q,q_{1}}I & = & \left(1-\frac{I^{*}}{I}\right)\left[\beta SI+\beta_{1}VI-\left(\mu +\gamma +d_{1}\right)I\right],\;\\ & = & \left(1-\frac{I^{*}}{I}\right)\left[\beta SI+\beta_{1}VI-\left(\frac{\beta S^{*}I^{*}+\beta_{1}V^{*}I^{*}}{I^{*}}\right)I\right],\;\\ & = & \beta S^{*}I^{*}\left(1-\frac{I}{I^{*}}-\frac{S}{S^{*}}+\frac{SI}{S^{*}I^{*}}\right)\;\\ & & +\beta_{1}V^{*}I^{*}\left(1-\frac{I}{I^{*}}-\frac{V}{V^{*}}+\frac{VI}{V^{*}I^{*}}\right). \end{array} \end{array}$$

Using Equations (16)–(18) in Equation (15), we have

(19) $$\begin{array}{c} \begin{array}{ccc} {}^{H}D_{0,\phi }^{q,q_{1}}L & = & -\mu S^{*}\left(\frac{S^{*}}{S}+\frac{S}{S^{*}}-2\right)-\beta S^{*}I^{*}\left(\frac{S^{*}}{S}+\frac{S}{S^{*}}-2\right)\;\\ & & -\omega S^{*}\left(\frac{S^{*}}{S}+\frac{V}{V^{*}}+\frac{SV^{*}}{VS^{*}}-3\right). \end{array} \end{array}$$

Therefore, ${}^{H}D_{0,\phi }^{q,q_{1}}L\left(t\right)\leq 0$ when $R_{0}^{v}>1$. It follows from Theorem 1 that the endemic equilibrium $P_{1}$ of the fractional-order model (6) when $R_{0}^{v}>1$ is globally asymptotically stable. □

### 2.8. Numerical Scheme and Its Results

This section discusses the numerical scheme for the new fractional generalized derivative and obtains numerical simulations. We follow the same stepping as mentioned in [41]. The generalized fractional derivative is given by

(20) $$\begin{array}{c} D_{0,\phi }^{q,q_{1}}y\left(t\right)=g\left(t,y\left(t\right)\right). \end{array}$$

Equation (20) is converted into the following form:

(21) $$\begin{array}{c} y\left(t\right)-\frac{y(0)\phi (0)}{\phi (t)}=\frac{1-q}{N(q)}g\left(t,y\left(t\right)\right)+\frac{q}{N\left(q\right)\Gamma \left(q_{1}\right)}\frac{1}{\phi (t)}\int_{0}^{t}{\left(t-\xi \right)}^{q_{1}-1}\phi \left(\xi \right)g\left(\xi ,y\left(\xi \right)\right)d\xi . \end{array}$$

Considering that $t_{n}=n\Delta t$, with n∈N, we have

(22) $$\begin{array}{c} \begin{array}{cc} y\left(t_{n+1}\right)= & \frac{y_{0}\phi \left(0\right)}{\phi \left(t_{n}\right)}+\frac{1-q}{N(q)}g\left(t_{n},y\left(t_{n}\right)\right)\\ & +\frac{q}{N\left(q\right)\Gamma \left(q_{1}\right)\phi \left(t_{n}\right)}\int_{0}^{t_{n+1}}{\left(t_{n+1}-\xi \right)}^{q_{1}-1}\phi \left(\xi \right)g\left(\xi ,y\left(\xi \right)\right)d\xi , \end{array} \end{array}$$

which leads to the following,

(23) $$\begin{array}{cc} y\left(t_{n+1}\right)= & \frac{y_{0}\phi \left(0\right)}{\phi \left(t_{n}\right)}+\frac{1-q}{N(q)}g\left(t_{n},y\left(t_{n}\right)\right)\\ \; & +\frac{q}{N\left(q\right)\Gamma \left(q_{1}\right)\phi \left(t_{n}\right)}\sum_{k=0}^{n}\int_{t_{k}}^{t_{k+1}}{\left(t_{n+1}-\xi \right)}^{q_{1}-1}l\left(\xi ,y\left(\xi \right)\right)d\xi , \end{array}$$

where l(ξ,y(ξ))=ϕ(ξ)g(ξ,y(ξ)). One can approximate the function *l* in the interval $\left[t_{k},t_{k+1}\right]$ as it is given in [42]. The Lagrange polynomial interpolation passing these points $\left(t_{k-1},l\left(t_{k-1},y_{k-1}\right)\right)$ and $\left(t_{k},l\left(t_{k},y_{k}\right)\right)$ is as follows:

(24) $$\begin{array}{c} \begin{array}{ccc} P_{k}\left(\xi \right) & = & \frac{\xi -t_{k}}{t_{k-1}-t_{k}}l\left(t_{k-1},y\left(t_{k-1}\right)\right)+\frac{\xi -t_{k-1}}{t_{k}-t_{k-1}}l\left(t_{k},y\left(t_{k}\right)\right)\;\\ & \approx & \frac{l(t_{k-1},y_{(}k-1))}{\Delta t}\left(t_{k}-\xi \right)+\frac{l(t_{k},y_{(}k))}{\Delta t}\left(\xi -t_{k-1}\right). \end{array} \end{array}$$

Thus,

(25) $$\begin{array}{c} \begin{array}{ccc} y(t_{n+1}) & = & \frac{y(0)\phi (0)}{\phi (t_{n})}+\frac{1-q}{N\left(q\right)\phi \left(t_{n}\right)}l\left(t_{n},y_{n}\right)\;\\ & & +\frac{q}{N\left(q\right)\Gamma \left(q_{1}\right)\phi \left(t_{n}\right)}\sum_{k=0}^{n}[\frac{l(t_{k},y_{k})}{\Delta t}\int_{t_{k}}^{t_{k+1}}\left(\xi -t_{k-1}\right){\left(t_{n+1}-\xi \right)}^{q_{1}-1}d\xi \;\\ & & +\frac{l(t_{k-1},y_{k-1})}{\Delta t}\int_{t_{k}}^{t_{k}+1}\left(\xi -t_{k}\right){\left(t_{n+1}-\xi \right)}^{q_{1}-1}d\xi ] \end{array} \end{array}$$

The integrals inside Equation (28) can be determined as follows:

(26) $$\begin{array}{c} \begin{array}{ccc} \int_{t_{k}}^{t_{k+1}}\left(\xi -t_{k-1}\right){\left(t_{n+1}-\xi \right)}^{q_{1}-1}d\xi & = & \frac{h^{q_{1}+1}}{q_{1}\left(q_{1}+1\right)}A_{n,k,q_{1}}^{1},\;\\ \int_{t_{k}}^{t_{k+1}}\left(t_{k}-\xi \right){\left(t_{n+1}-\xi \right)}^{q_{1}-1}d\xi & = & \frac{h^{q_{1}+1}}{q_{1}\left(q_{1}+1\right)}A_{n,k,q_{1}}^{2} \end{array} \end{array}$$

where

(27) $$\begin{array}{c} \begin{array}{c} A_{n,k,q_{1}}^{1}=\left[{\left(n-k+1\right)}^{q_{1}}\left(n-k+2+q_{1}\right)-{\left(n-k\right)}^{q_{1}}\left(n-k+2+2q_{1}\right)\right],\;\\ A_{n,k,q_{1}}^{2}=\left[{\left(n-k\right)}^{q_{1}}\left(n-k+1+q_{1}\right)-{\left(n-k+1\right)}^{q_{1}+1}\right], \end{array} \end{array}$$

Finally, we achieve the required scheme as follows:

(28) $$\begin{array}{c} \begin{array}{ccc} y(t_{n+1}) & = & \frac{y(0)\phi (0)}{\phi (t_{n})}+\frac{1-q}{N\left(q\right)\phi \left(t_{n}\right)}g\left(t_{n},y_{n}\right)\;\\ & & +\frac{qh^{q_{1}}}{N\left(q\right)\Gamma \left(q_{1}+2\right)\phi \left(t_{n}\right)}\sum_{k=0}^{n}[\phi \left(t_{k}\right)g\left(t_{k},y_{k}\right)A_{n,k,q_{1}}^{1}\;\\ & & +\phi \left(t_{k-1}\right)g\left(t_{k-1},y_{k-1}\right)A_{n,k,q_{1}}^{2}] \end{array} \end{array}$$

### 2.9. Sensitivity Analysis

Sensitivity analysis is critical for determining how best to minimize coronavirus mortality and morbidity, as well as the relative relevance of the many factors responsible for its transmission and prevalence. In this subsection, we will find the model parameters that have a large influence on $R_{0}^{v}$. The following formula should be used to determine the sensitivity analysis of the parameters involved in the basic reproduction number $R_{0}^{v}$ [43].

**Definition** **3.** *The normalized forward sensitivity index of a variable, w, for which differentiability depends on a parameter, q, is defined as*

(29) $$\begin{array}{c} \prod_{q}^{w}:=\frac{\partial w}{\partial q}\times \frac{q}{w}. \end{array}$$

Using the formula mentioned in the above definitions, we calculate the analytical expression of $R_{0}^{v}$, $\prod_{q}^{R_{0}^{v}}:=\frac{\partial R_{0}^{v}}{\partial p}\times \frac{q}{R_{0}^{v}}$ for each of the different parameters β=0.28, $\beta_{1}=0.2$, γ=0.05, $\gamma_{1}=0.04$, μ=1/(74.87×365), $d_{1}=0.024$, and ω=0.15. We now calculate the sensitivity index of $R_{0}^{v}$ with respect to the parameter β as

$$\begin{array}{c} \prod_{\beta }^{R_{0}^{v}}:=\frac{\partial R_{0}^{v}}{\partial \beta }\times \frac{\beta }{R_{0}^{v}}=0.272026. \end{array}$$

In a similar way, we can calculate the rest of the indices as shown in Table 1.

It can be observedfrom the values given in Table 1 that the parameter $\beta_{1}$, followed by γ, $d_{1}$, β, and so on, can increase or decrease the basic reproduction number. Upon decreasing the contact among the susceptible and infected, and increasing the recovery rate by vaccinating the individuals, the number of infected individuals shall decrease.

## 3. Results and Discussion

**Figure 2:**
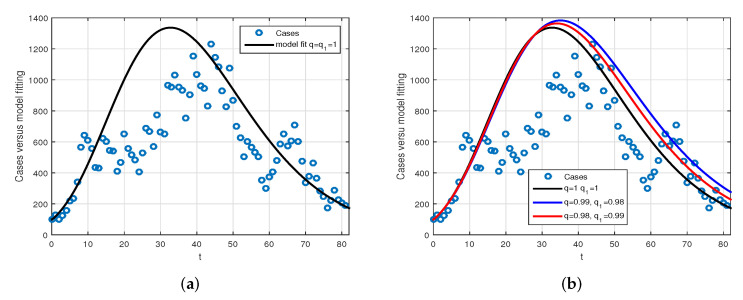
The data versus the model fitting for the cases 1 May–4 August 2022. The circle denotes real data while the bold curve denotes the model solution. Subfigure (**a**) represents the comparison of data versus model when $q=q_{1}=1$, while subfigure (**b**) is the comparison of data versus model for various values of *q* and $q_{1}$.

**Figure 3:**
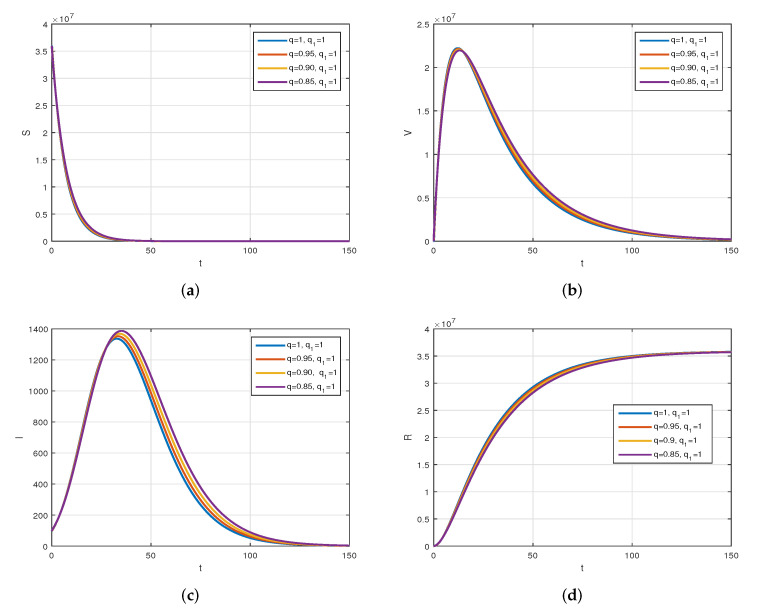
The plot displays the behavior of the model for various values of *q* and $q_{1}=1$. Subfigure (**a**) shows the simulation of healthy population when varying *q* and keeping $q_{1}$ fixed. Subfigure (**b**) is the simulation of vaccinated population when $q_{1}=1$ is fixed and varying *q*. Subfigure (**c**) shows the simulation of infected population for various values of *q* and fixed $q_{1}$. Subfigure (**d**) is the simulation of recovered individuals when varying *q* and fixing $q_{1}$.

**Figure 4:**
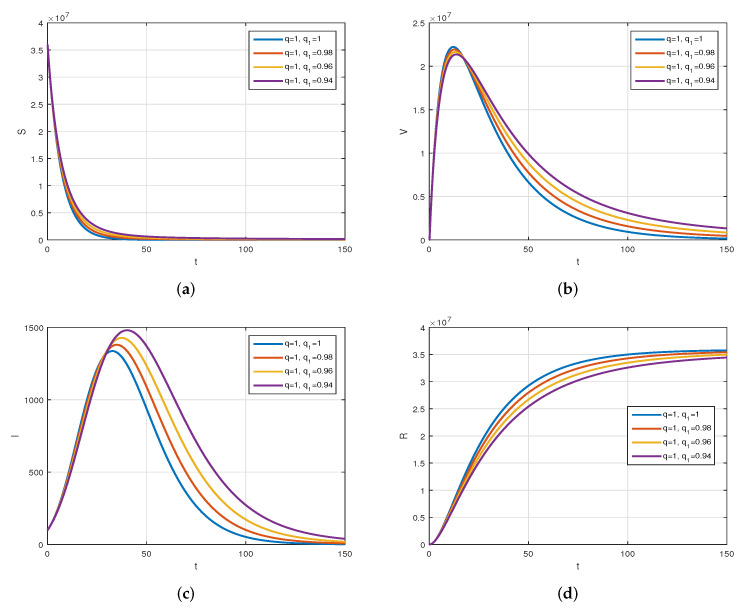
The plot display the behavior of the model for various values of $q_{1}$ and q=1. Subfigure (**a**) shows the simulation of healthy population when fixing *q* and varying $q_{1}$. Subfigure (**b**) shows the simulation of vaccinated population when fixing *q* and varying $q_{1}$. Subfigure (**c**) shows the simulation of infected population when fixing *q* and varying $q_{1}$. Subfigure (**d**) shows the simulation of recovered population when fixing *q* and varying $q_{1}$.

**Figure 5:**
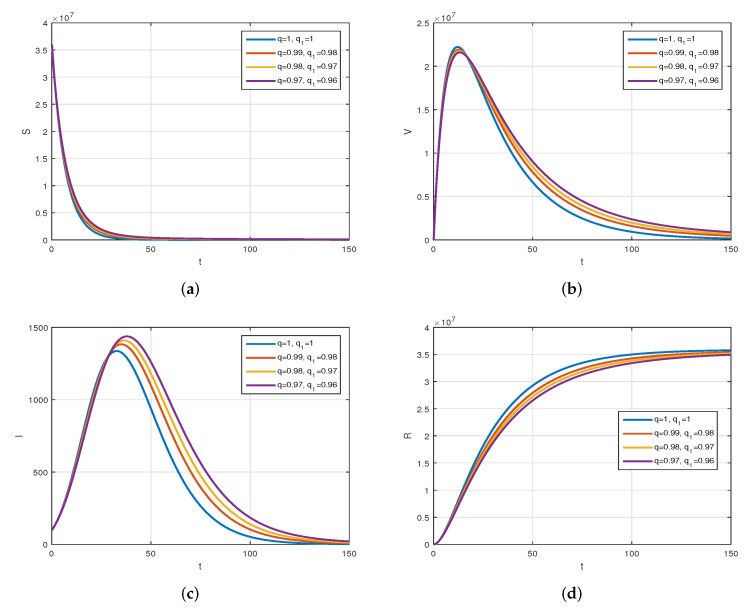
The plot displays the behavior of the model for various values of *q* and $q_{1}$. Subfigure (**a**) shows the simulation of healthy population for various values of *q* and $q_{1}$. Subfigure (**b**) shows the simulation of vaccinated population for various values of *q* and $q_{1}$. Subfigure (**c**) shows the simulation of infected population for various values of *q* and $q_{1}$. Subfigure (**d**) shows the simulation of recovered population for various values of *q* and $q_{1}$.

**Figure 6:**
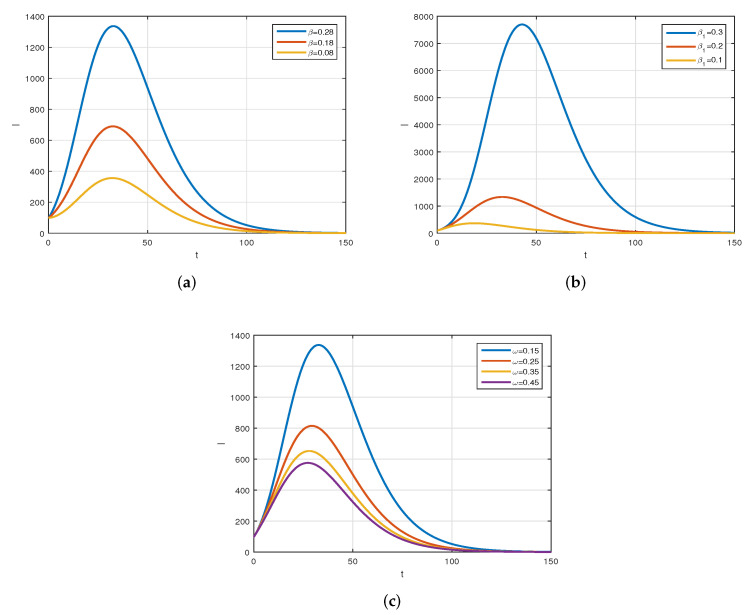
The behavior of the infected population for different values of β, $\beta_{1}$, and ω. Subfigure (**a**) shows different values of β, while Subfigure (**b**) is given for various values of $\beta_{1}$, and (**c**) is given for various values of ω.

**Figure 7:**
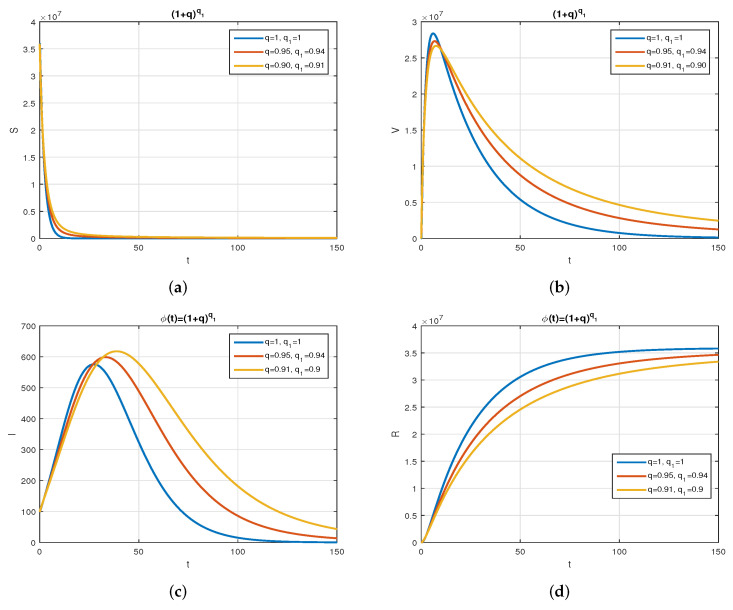
The behavior of the model for different values of *q* and $q_{1}$ with $\phi \left(t\right)={\left(1+q\right)}^{q_{1}}$. Subfigure (**a**) shows the simulation of the healthy population for various values of *q* and $q_{1}$ with $\phi \left(t\right)={\left(1+q\right)}_{1}^{q}$. Subfigure (**b**) shows the simulation of the vaccinated population for various values of *q* and $q_{1}$ with $\phi \left(t\right)={\left(1+q\right)}_{1}^{q}$. Subfigure (**c**) shows the simulation of the infected population for various values of *q* and $q_{1}$ with $\phi \left(t\right)={\left(1+q\right)}_{1}^{q}$. Subfigure (**d**) shows the simulation of the recovered population for various values of *q* and $q_{1}$ with $\phi \left(t\right)={\left(1+q\right)}_{1}^{q}$.

**Figure 8:**
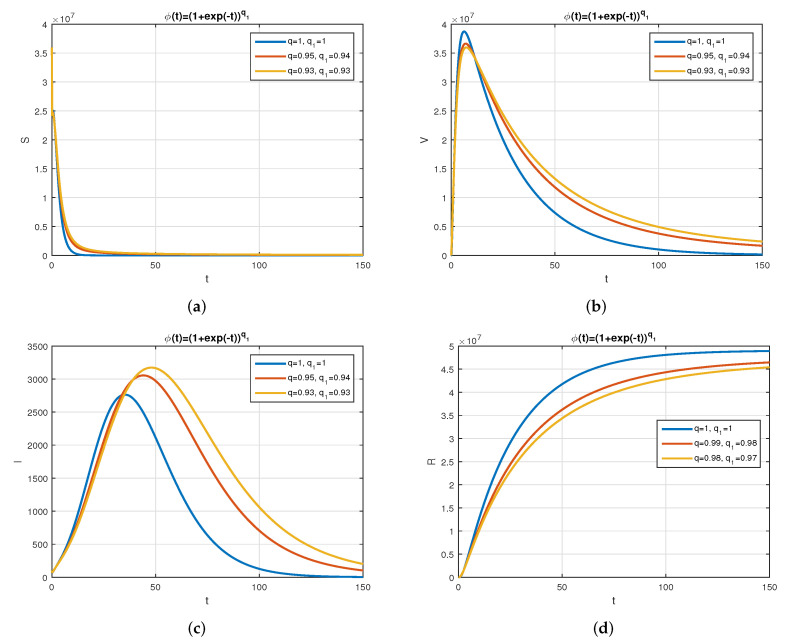
The behavior of the model for different values of *q* and $q_{1}$ with $\phi \left(t\right)={\left(1+\exp\left(-t\right)\right)}^{q_{1}}$. Subfigure (**a**) shows the simulation of healthy population for various values of *q* and $q_{1}$ with $\phi \left(t\right)={\left(1+\exp\left(-t\right)\right)}^{q_{1}}$. Subfigure (**b**) shows the simulation of vaccinated population for various values of *q* and $q_{1}$ with $\phi \left(t\right)={\left(1+\exp\left(-t\right)\right)}^{q_{1}}$. Subfigure (**c**) shows the simulation of infected population for various values of *q* and $q_{1}$ with $\phi \left(t\right)={\left(1+\exp\left(-t\right)\right)}^{q_{1}}$. Subfigure (**d**) shows the simulation of recovered population for various values of *q* and $q_{1}$ with $\phi \left(t\right)={\left(1+\exp\left(-t\right)\right)}^{q_{1}}$.

**Figure 9:**
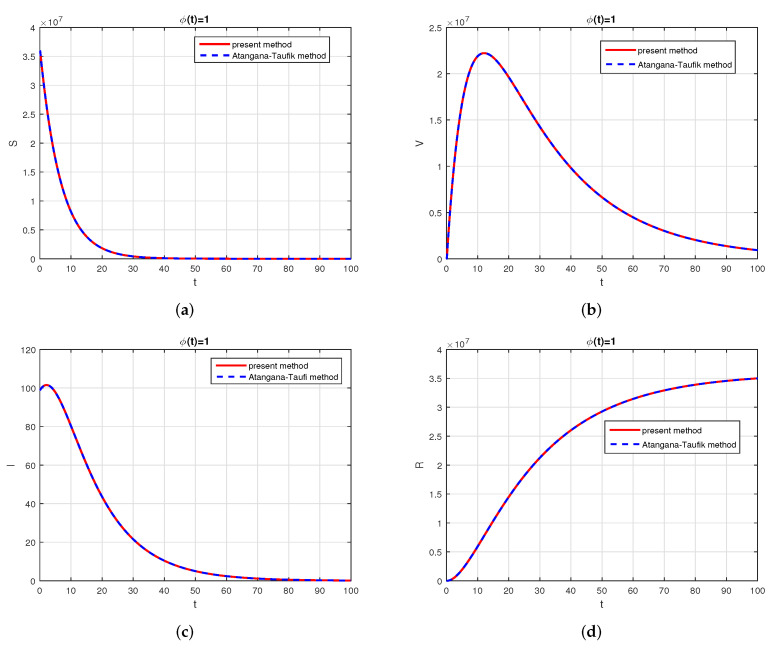
Comparison of the present method with Atangana–Taufik method, for q=1, $q_{1}=1$, and ϕ(t)=1. Subfigure (**a**) shows the comparison of the schemes for healthy population when ϕ(t)=1 and $q=q_{1}=1$. Subfigure (**b**) shows the comparison of the schemes for vaccinated population when ϕ(t)=1 and $q=q_{1}=1$. Subfigure (**c**) shows the comparison of the schemes for infected population when ϕ(t)=1 and $q=q_{1}=1$. Subfigure (**d**) shows the comparison of the schemes for recovered population when ϕ(t)=1 and $q=q_{1}=1$.

**Figure 10:**
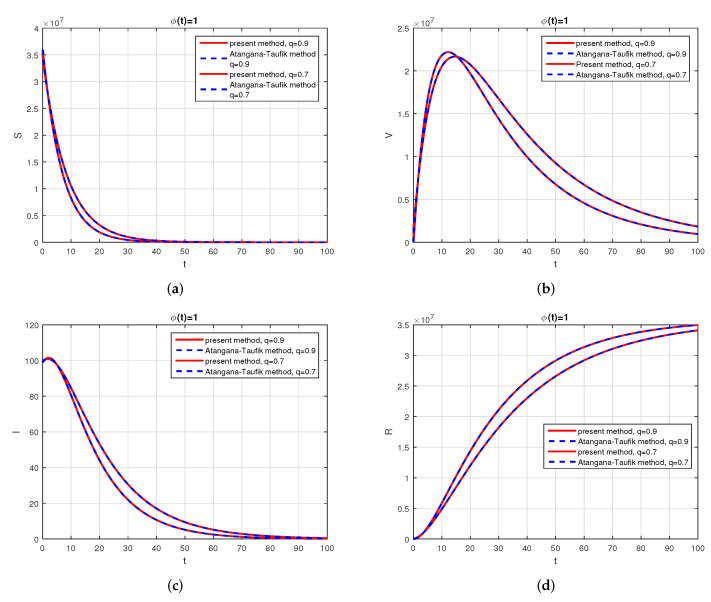
Comparison of the present method with Atangana–Taufik method, for q=0.9,0.7, $q_{1}=1$ and ϕ(t)=1. Subfigure (**a**) shows the comparison of the schemes for healthy population when ϕ(t)=1 and q=0.9,0.7, $q_{1}=1$. Subfigure (**b**) shows the comparison of the schemes for vaccinated population when ϕ(t)=1 and q=0.9,0.7, $q_{1}=1$. Subfigure (**c**) shows the comparison of the schemes for infected population when ϕ(t)=1 and q=0.9,0.7, $q_{1}=1$. Subfigure (**d**) shows the comparison of the schemes for recovered population when ϕ(t)=1 and q=0.9,0.7, $q_{1}=1$.

**Figure 11:**
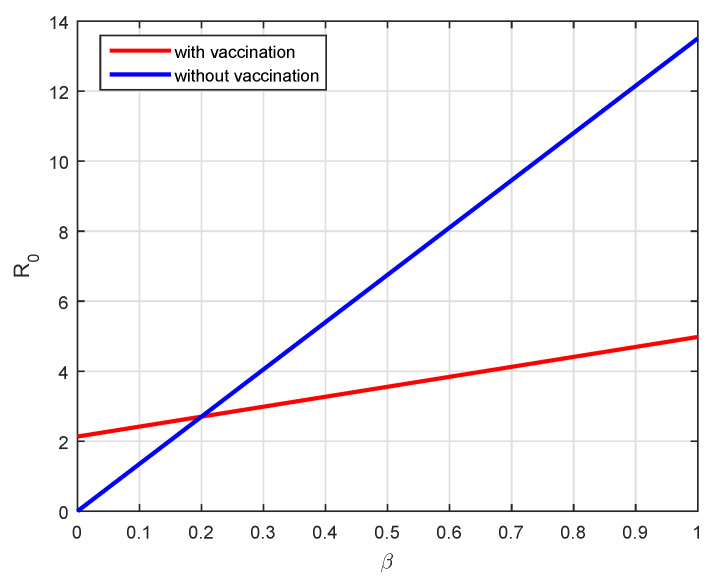
Comparison of the basic reproduction number with vaccination and without vaccination.

We consider the following numerical values and the initial conditions in our numerical simulation of the model (6): Λ=1273.94, β=0.28, $\beta_{1}=0.2$, γ=0.05, $\gamma_{1}=0.04$, μ=1/(74.87×365), $d_{1}=0.024$, and ω=0.15, while S(0)=35942012, I(0)=99, V(0)=10, and R(0)=0. Using the real cases observed in Saudi Arabia, we plotted the model versus the data and obtained the results graphically in Figure 2. The cases have been given for the period (1 May–4 August 2022) and are the recently reported cases in the country. In Figure 2a the data are fitted to the model when ϕ(t)=1, $q=q_{1}=1$, while Figure 2b is obtained for different values of *q* and $q_{1}$. It can be observed that the behavior of the data show some good agreement with the model for the fractional case. Figure 3 shows the behavior of the model variables for various values of *q* and keeping $q_{1}$ fixed. Figure 4 is given to show the behavior of the model when *q* is fixed and $q_{1}$ varies for various values. Varying both the values of *q* and $q_{1}$, we have plotted the results graphically in Figure 5. The contact rates β and $\beta_{1}$ have a great impact on the disease spread and control, which is shown graphically in Figure 6. When the value of β (the contact among the healthy and the infected compartments, such as social distancing, avoiding gatherings, using face masks, etc.) and $\beta_{1}$ (the contact among healthy and vaccinated people) decrease, the number of infected people decreases. We give the results of the model numerically in Figure 7 and Figure 8 when $\phi \left(t\right)={\left(1+q\right)}^{q_{1}}$ $\phi \left(t\right)={\left(1+\exp\left(-t\right)\right)}^{q_{1}}$, respectively, for many values of the fractional-order parameters *q* and $q_{1}$. One of the advantages of this new fractional derivative is the use of the function ϕ(t), where one can fit the data well using an appropriate value for ϕ(t). We also compare the present scheme given in [41] with [42]. We provide such a comparison in Figure 9 and Figure 10. In Figure 9, we fix ϕ(t)=1, $q=q_{1}=1$ and show the comparison of the present method with the Atangana–Taufik scheme shown in [42]. Similarly, when q=0.9,0.7, we show the comparison of the results in Figure 10. It should be noted that the present scheme generalizes the Atangana–Taufik method, so we put ϕ(t)=1. The comparison of the basic reproduction number with and without vaccination is shown in Figure 11. It is clear from the comparison results that the present scheme is matched perfectly with the scheme given in [42].

## 4. Conclusions

We investigated the dynamics of coronavirus infection cases from Saudia Arabia using the fractional model. The new fractional derivative considered was recently reported in the literature and is known as the generalized Hattaf fractional derivative. We presented the background results for the new fractional derivative and then considered the model using the Hattaf derivative. The equilibrium points are obtained, and their stability is discussed. It can be observed that the fractional model is locally asymptotically stable when $R_{0}<1$, and it is unstable otherwise. Further, we obtain the global stability of the model when the basic reproduction number $R_{0}>1$. Considering the reported cases of the COVID-19 in Saudi Arabia, the model is fitted to the data, and their results are obtained for both integer and non-integer cases. For the behaviors of the fractional-order parameters *q* and $q_{1}$, we have presented some numerical results that demonstrate the effectiveness of the new fractional derivative. The basic reproduction number without vaccination is $R_{0}=3.78$, while with vaccination, it is $R_{0}^{v}=2.92$. One of this generalized fractional derivatives is the use of the new function that results when dealing with data of a different nature, which are difficult to fit using other fractional operators. The parameters’ values that decrease the future cases have been shown graphically.

## Figures and Tables

**Table 1:** Sensitivity indices of $R_{0}^{v}$ associated to their parameters.

Parameter	Sensitivity Index
β	0.272026
$\beta_{1}$	0.727974
ω	−0.0613474
$\gamma_{1}$	0.0612913
μ	−0.000438186
γ	−0.675342
$d_{1}$	−0.324164

## Data Availability

Data used in the manuscript are available from the corresponding author upon reasonable request.
